# The effect of action-based entrepreneurship education on intention to become an entrepreneur

**DOI:** 10.1016/j.mex.2022.101657

**Published:** 2022-03-08

**Authors:** Omar Boubker, Khaled Naoui, Abdelaziz Ouajdouni, Maryem Arroud

**Affiliations:** a Laayoune Higher School of Technology, Ibn Zohr University, Morocco; b National School of Business and Management, Dakhla, Ibn Zohr University, Morocco; c National School of Business and Management, Abdelmalek Essaadi University, Tangier, Morocco

**Keywords:** Learning-by-doing, Entrepreneurship, Public university students, entrepreneurial intention

## Abstract

The kingdom of Morocco has launched over the last decade major reform projects in order to strengthen youth entrepreneurship. Therefore, it is important to identify factors contributing to enhanced youth entrepreneurship activity. Hence, this method article examines the determinants of public university students’ entrepreneurial intention, by focusing on the importance of action-based entrepreneurship education. Data were collected using a face-to-face questionnaire from management students who had completed a program in action-based entrepreneurship. The data analysis design incorporates both exploratory (PCA using IBM SPSS Statistics 26) and confirmatory factor analysis (PLS-SEM using SmartPLS 3). Findings showed that action-based entrepreneurship education positively and significantly affects attitude towards entrepreneurship, and perceived entrepreneurial capacity. In addition, social norms positively influence attitude towards entrepreneurship and perceived entrepreneurial capacity, which turns to enhance students' entrepreneurial intention. Managers of Moroccan higher schools of technology may use this method article to pinpoint critical factors for enhancing students' entrepreneurial intention.

• This method article proposes a practical approach to teaching entrepreneurship based on the learning-by-doing approach.
• This method article can be used as a reference for researchers interested in studying the role of entrepreneurship education in promoting entrepreneurship in universities.
• This method article can be used in order to identify the determinants of entrepreneurial intent among engineering students.

## Graphical abstract

**Figure:**
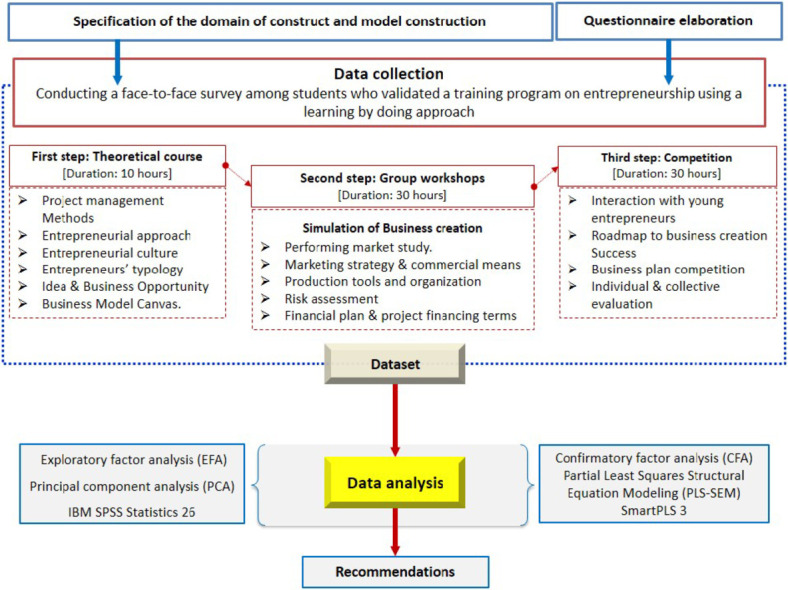

Specifications Table

**Table:** 

Subject Area:	Environmental Science
More specific subject area:	Entrepreneurship
Method name:	Evaluate the effect of entrepreneurship education on intention to become an entrepreneur using the exploratory factor (PCA) and confirmatory factor analysis (PLS-SEM).
Name and reference of original method:	Exploratory factor analysis [1] and partial least squares structural equation modeling [2].
Resource availability:	Repository name: Mendeley Data DOI:10.17632/fp8f4d8djy.4 Direct URL to data: https://data.mendeley.com/datasets/fp8f4d8djy/4
Related research article:	O. Boubker, M. Arroud, A. Ouajdouni, Entrepreneurship education versus management students’ entrepreneurial intentions. A PLS-SEM approach, *Int. J. Manag. Educ*. 19 (2021) 100450. 10.1016/j.ijme.2020.100450

## Method details

**Fig. 1:**
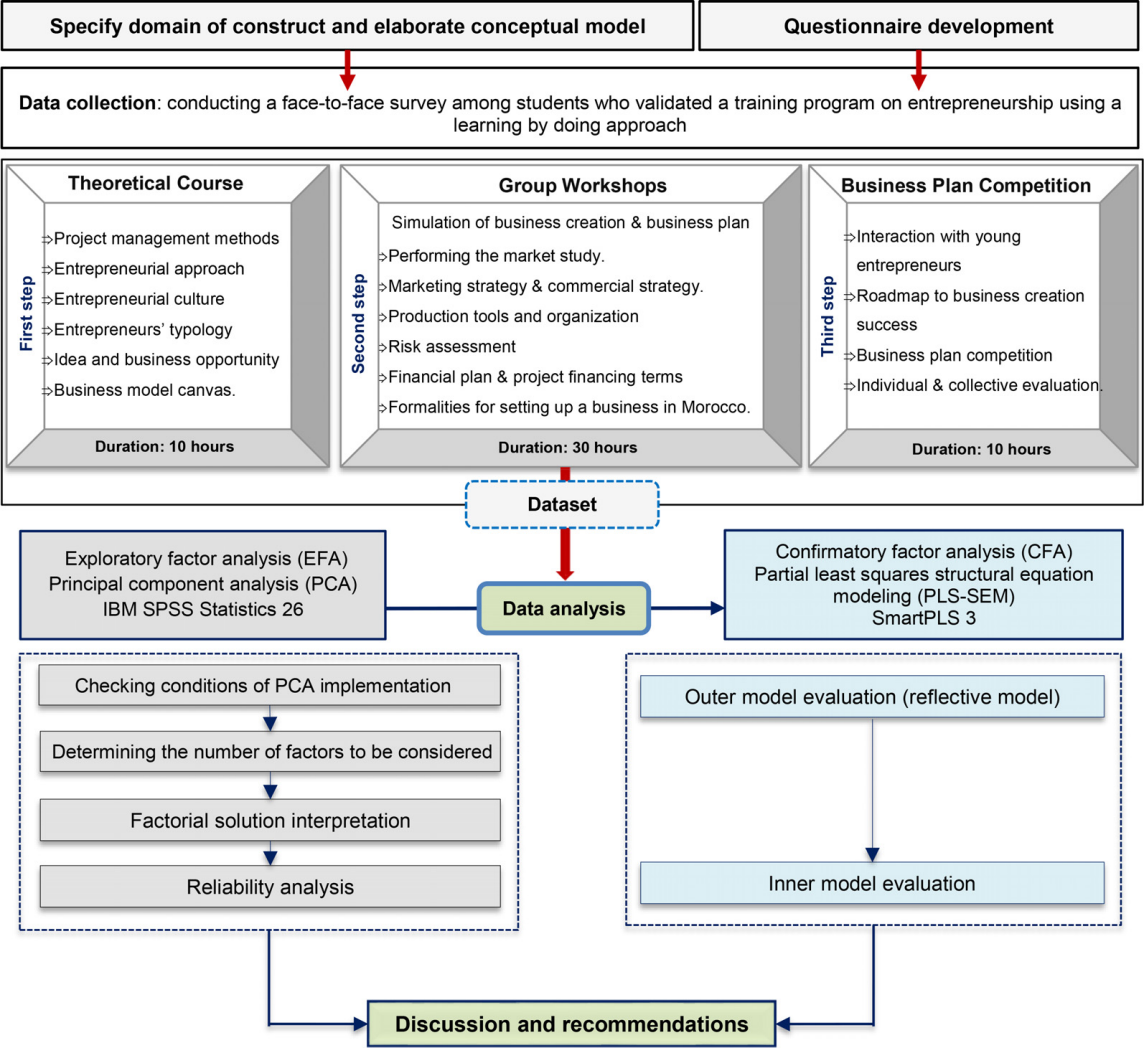
Proposed methodology steps.

With the aim of evaluating the effect of action-based entrepreneurship education on students' attitude towards entrepreneurship, perceived entrepreneurial capacity, and students' intentions to start up a business, this study mobilizes the exploratory factor analysis and the confirmatory factor analysis as two complementary approaches [1]. Fig. 1 outputs the different steps of the method implementation.

Table 1 synthesizes the different steps of setting up the exploratory and confirmatory factorial analysis. We performed principal component analysis (PCA) to purify the measurement scales. Further, we performed a structural equation modeling (SEM) to test hypotheses and the research model.

**Table 1:** Data analysis steps.

Steps	Criteria	Accepted value	
**First stage. Principal component analysis**			
Checking conditions of PCA implementation	Bartlett's Sphericity Test	p < 0.05	
	Kaiser-Meyer-Oklin (KMO)	KMO < 0.5	Unacceptable
		0.5 < KMO < 0.6	Miserable
		0.6 < KMO < 0.7	Mediocre
		0.7< KMO < 0.8	Middling
		0.8< KMO < 0.9	Meritorious
		KMO > 0.9	Marvelous
Determining the number of factors to be considered	Kaiser criterion	% total variance explained > 60%	
	Examination of eigenvalues	Selection the factors before inflection point	
Factorial solution interpretation	Varimax rotation - Orthogonal rotation: in order to streamline interpretation of the factors by reducing the number of variables with strong correlations on each factorial axis.		
	Communalities	value must be higher than 0.4	
	Factor loading	value must be higher than 0.5	
Reliability analysis	Crombach alpha	α ≥ 0.60	
**Second stage. CFA -Partial least squares structural equation modeling (PLS-SEM)**			
A **Outer model evaluation (reflective model)**			
Convergent validity assessment	Cronbach's alpha	α value must be higher than 0.7	
	Reliability	ρ_A_ value must be higher than 0.7	
	Composite reliability	ρc value must be higher than 0.7	
	Loadings	Loadings must be higher than 0.7	
	Average variance extracted	AVE must be higher than 0.5	
Discriminant Validity assessment	Cross-loadings	The loading of an indicator on its assigned latent variable should be higher than its loadings on all other variables.	
	Heterotrait-Monotrait Ratio	The HTMT ratio values must be lower than 0.9	
	Fornell-Larcker criterion	The square root of the AVEs for each structure should be greater than the construct's correlations with all other constructs	
A **Second stage: Inner model evaluation**			
Endogenous latent variables coefficient of determination	R² < 0.19	Unacceptable	
	0.19 ≤ R² < 0.33	Weak	
	0.33 ≤ R² < 0.67	Moderate	
	R² ≥ 0.67	Substantial	
Effect size	f^2^ < 0.02	No effect size	
	0.02 ≤ f^2^ < 0.15	Small	
	0.15 ≤ f^2^ < 0.35	Moderate	
	f^2^ ≥ 0.35	Large	
Predictive relevance	Q Square	Q^2^ must be higher than 0	
Goodness-of-fit	GoF < 0.10	No fit	
	0.1 ≤ GoF < 0.25	Small	
	0.25 ≤ GoF < 0.36	Medium	
	GoF ≥ 0.36	Large	
Hypotheses testing	t-value = 1.96	Significant at p-value <0.05*	
	t-value = 2.58	Significant at p-value < 0.01**	
	t-value = 3.29	Significant at p-value < 0.001***.	

### Conceptual model

**Fig. 2:**
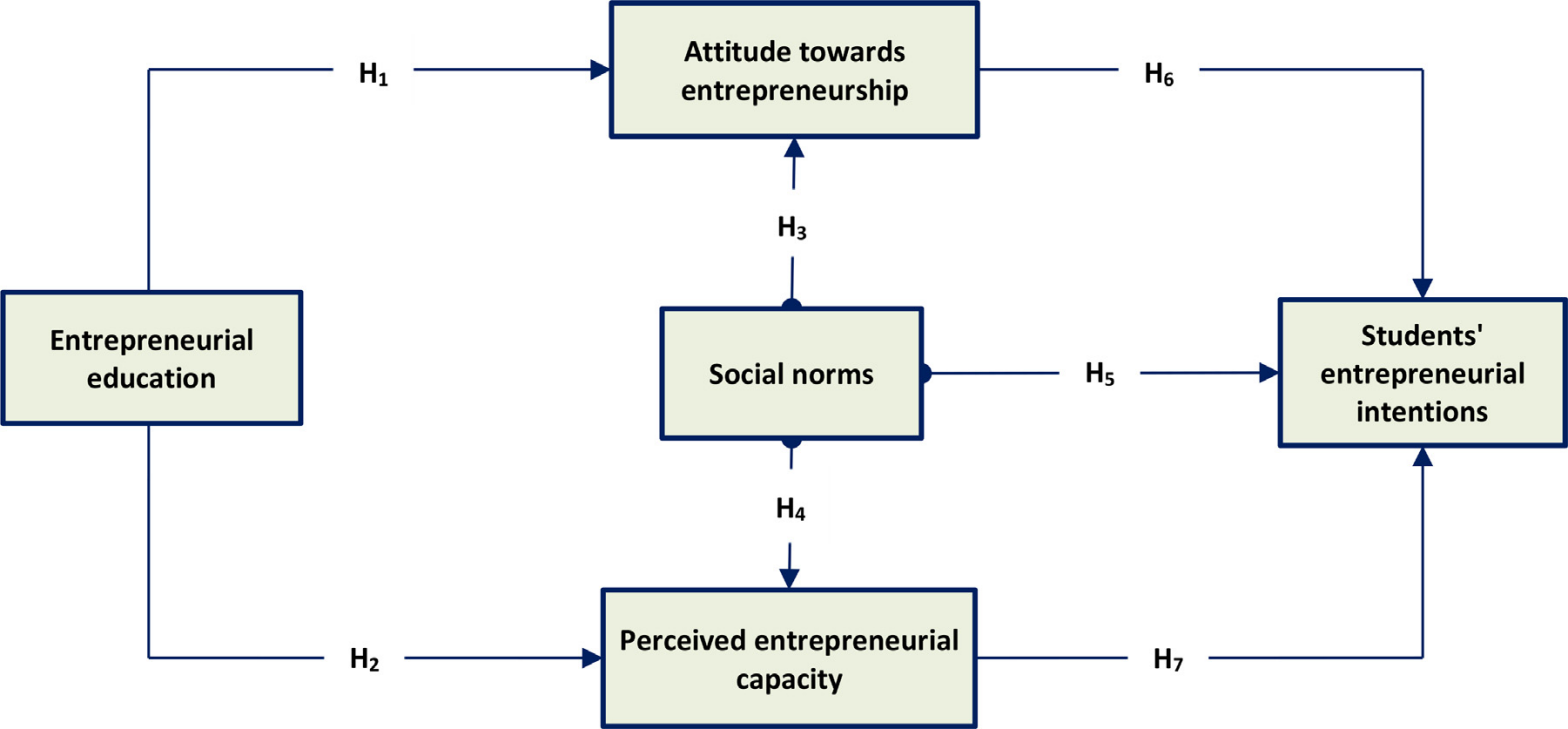
Research model.

The conceptual model of this study was built on the expansion of the theory of planned behavior, by adding entrepreneurship education. Fig. 2 outlines the conceptual model, which supposes the direct and positive effect of entrepreneurial education on attitude towards entrepreneurship (H_1_), and perceived entrepreneurial capacity (H_2_). This model also indicates that social norms influence attitude towards entrepreneurship (H_3_), perceived entrepreneurial capacity (H_4_), and students' entrepreneurial intentions (H_5_). In addition, attitude towards entrepreneurship (H_6_), and perceived entrepreneurial capacity (H_7_) positively influence students' entrepreneurial intentions.

### Constructs operationalization

For operationalization of the constructs, we used measurement scales selected from existing studies. Therefore, attitude towards entrepreneurship (ATE) was measured with five items [3]. We selected four items to measure social norms (SON) [4]. The measurement scale for perception of entrepreneurial capacity (ENC) comprised 14 items [5]. The students’ entrepreneurial intentions were measured using six items [6]. Entrepreneurship education (ENE) was measured using eight items [7]. As well, a 7-item Likert-type scale ranging from 1 (total disagreement) to 7 (total agreement) was employed to measure the questions related to these variables.

The sampling frame consisted of final-year management students of Laayoune Higher School of Technology, including professional bachelor and university diploma of technology students.

These students underwent 50 hours of entrepreneurship and project management education. At this level, the pedagogical program adopted was designed around the learning by doing approach, which was conducted in three steps. The first step provided students with a theoretical background of entrepreneurship, by focusing on project management methods, entrepreneurial approach, entrepreneurial culture, entrepreneurs’ typology, idea and business opportunity, and business model canvas. The second step consisted of in-group workshops composed of five students, working together on a business idea, market study, and the elaboration of the financial plan. After this second step, the last step consisted in organizing a business plan competition in order to conduct an individual and collective evaluation. This training program is designed to build a positive attitude among management students in terms of self-efficacy and tolerance for ambiguity, as well as to improve their knowledge and skills, particularly in marketing, finance, problem-solving and critical thinking [8].

### Data collection technique

**Fig. 3:**
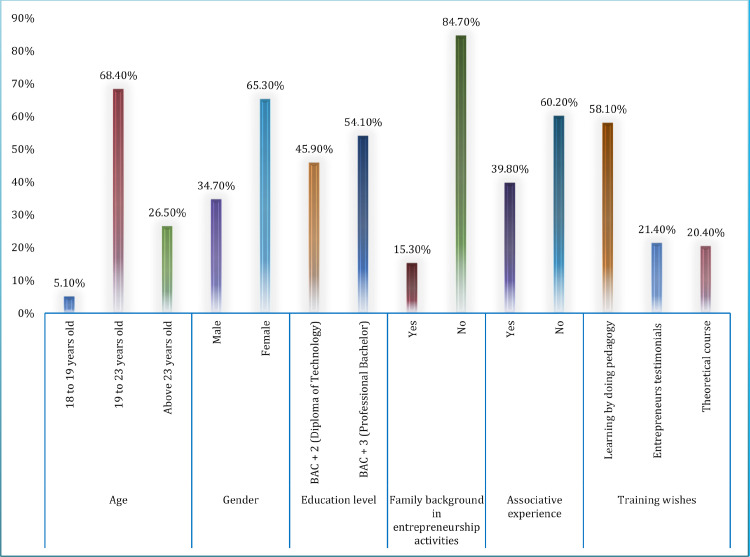
Socio-demographic characteristics of the surveyed students.

The questionnaire was conducted face-to-face among students who validated this training program, during a week-long period in April 2019. At this stage, 98 eligible responses have been obtained. As illustrated in Fig. 3, the sample included more females (65.3%) than males (34.7%), with the majority of them are aged between 19 and 23 years (68.4%). More than 54 percent of participants in this survey were students of the professional bachelor's degree in human resources management, whereas 45.9 percent of them were studying for a university diploma of technology in management techniques. Further, 58 percent of the interviewed students prefer entrepreneurship training based on the learning by doing approach. The largest proportion of surveyed students (84.7%) had no family background in entrepreneurial activities. Lastly, only 39.8 percent of them have previously been volunteers with associations.

## Finding and discussions

### PCA results and discussion

The implementation of the principal component analysis (PCA) procedure allowed the purification of the different measurement scales. Using the IBM SPSS Statistics 26, this technique allowed us to remove ten items serving to measure the perceived entrepreneurial capacity, including ENC2, ENC3, ENC4, ENC6, ENC8, ENC9, ENC11, ENC12, ENC13, and ENC14. These items showed low scores regarding commonality (< 0.4) and loading (< 0.5). In addition, the PCA indicated that for each of the measurement scales only a single factor was retained (Table 2).

**Table 2:** Results of measurement scale purification using principal component analysis technique.

Construct	Items	KMO and Bartlett's Test				Communalities	Loading	Reliability(α)	Total variance explained
		KMO	Approx. Chi-Square	df	Sig.				
Entrepreneurial education (8 items)	ENE1	.855	542.537	28	.000	.645	.803	.916	63.37%
	ENE2					.669	.818		
	ENE3					.496	.704		
	ENE4					.654	.809		
	ENE5					.615	.784		
	ENE6					.735	.858		
	ENE7					.629	.793		
	ENE8					.626	.791		
Attitude towards entrepreneurship (5 items)	ATE1	.874	390.472	10	.000	.625	.791	.923	77.27%
	ATE2					.757	.870		
	ATE3					.850	.922		
	ATE4					.800	.894		
	ATE5					.832	.912		
Social norms (4 items)	SON1	.746	101.615	6	.000	.506	.711	.767	59.35 %
	SON2					.688	.830		
	SON3					.658	.811		
	SON4					.522	.723		
Perceived entrepreneurial capacity (4 items)	ENC1	.771	104.206	6	.000	.609	.780	.783	60.80%
	ENC5					.666	.816		
	ENC7					.594	.771		
	ENC10					.563	.750		
Students’ entrepreneurial intentions (6 items)	SEI1	.869	427.066	15	.000	.658	.811	.914	70.61%
	SEI2					.783	.885		
	SEI3					.883	.940		
	SEI4					.749	.866		
	SEI5					.551	.742		
	SEI6					.612	.782		

Extraction Method: Principal Component Analysis.

### PLS-SEM results and discussion

Table 3 presents the evaluation of the reflective measurement models. The average variance extracted, the Cronbach's alpha, the reliability (ρ_A_), and the composite reliability (ρ_c_) values are higher than 0.5, 0.7, 0.7, and 0.7, respectively. Moreover, discriminant validity is checked using the Fornell-Larcker criterion [9], and the Heterotrait-Monotrait (HTMT) ratio [10]. Likewise, the discriminant validity was assessed according to the cross-loading (Table 4).

**Table 3:** Assessment of constructs reliability and validity.

Latent variable	Convergence validity				Fornell-Larcker criterion.					HTMT criterion.				
	AVE	α	ρ_A_	ρ_c_	1	2	3	4	5	1	2	3	4	5
**1. ATE**	0.77	0.93	0.93	0.94	**0.88**									
**2. ENE**	0.63	0.92	0.92	0.93	0.54	**0.80**				0.58				
**3. ENC**	0.61	0.78	0.79	0.86	0.58	0.53	**0.78**			0.68	0.61			
**4. SON**	0.59	0.77	0.78	0.85	0.61	0.52	0.57	**0.77**		0.70	0.61	0.71		
**5. SEI**	0.71	0.92	0.92	0.93	0.66	0.62	0.52	0.43	**0.84**	**0.71**	0.68	0.61	0.51

**Table 4:** Assessment of constructs discriminant validity using cross loading.

	ATE	ENE	ENC	SON	SEI
ATE1	**0.79**	0.45	0.48	0.52	0.41
ATE2	**0.88**	0.52	0.56	0.55	0.64
ATE3	**0.92**	0.49	0.53	0.56	0.60
ATE4	**0.89**	0.42	0.45	0.55	0.60
ATE5	**0.91**	0.48	0.54	0.47	0.60
ENE1	0.50	**0.81**	0.47	0.49	0.53
ENE2	0.43	**0.82**	0.47	0.46	0.54
ENE3	0.33	**0.69**	0.27	0.40	0.43
ENE4	0.45	**0.81**	0.42	0.39	0.43
ENE5	0.39	**0.78**	0.35	0.32	0.46
ENE6	0.45	**0.86**	0.50	0.45	0.54
ENE7	0.43	**0.80**	0.41	0.39	0.48
ENE8	0.42	**0.79**	0.44	0.37	0.55
ENC1	0.57	0.45	**0.80**	0.46	0.47
ENC5	0.44	0.45	**0.81**	0.43	0.45
ENC7	0.39	0.36	**0.75**	0.44	0.34
ENC10	0.41	0.39	**0.75**	0.44	0.37
SON1	0.50	0.41	0.51	**0.76**	0.38
SON2	0.41	0.43	0.41	**0.79**	0.33
SON3	0.36	0.34	0.27	**0.75**	0.28
SON4	0.55	0.39	0.49	**0.76**	0.31
SEI1	0.60	0.48	0.43	0.49	**0.82**
SEI2	0.58	0.55	0.49	0.28	**0.89**
SEI3	0.61	0.59	0.53	0.39	**0.94**
SEI4	0.53	0.49	0.41	0.29	**0.86**
SEI5	0.52	0.53	0.33	0.36	**0.74**
SEI6	0.46	0.51	0.43	0.36	**0.78**

Table 5 shows the results of inner model assessment based on the coefficient of determination (R^2^), and the predictive relevance (Q^2^). The R^2^ value of students' entrepreneurial intentions, attitude towards entrepreneurship, and perceived entrepreneurial capacity are 0.46; 0.44 and 0.40, respectively. Also, the data analysis indicates that the Q square values of all endogenous constructs are above 0, which demonstrates an acceptable predictive relevance [11].

**Table 5:** Inner model assessment based on R^2^ and Q^2^.

Latent variable	R Square	R Square Adjusted	Q Square
ATE	0.44	0.42	0.324
ENC	0.40	0.39	0.220
SEI	0.46	0.45	0.317

As shown in Table 6, all effect size values of exogenous construct on endogenous construct are acceptable, except the f^2^ value of social norms on students’ entrepreneurial intentions, which is 0.001.

**Table 6:** Inner model assessment based on the effect size values.

Exogenous construct		Endogenous construct	F Square value	Signification
ENE	→	ATE	0.123	Small effect size
ENE	→	ENC	0.129	Small effect size
SON	→	ATE	0.260	Moderate effect size
SON	→	ENC	0.196	Moderate effect size
SON	→	SEI	0.001	No effect size
ATE	→	SEI	0.300	Moderate effect size
ENC	→	SEI	0.054	Small effect size

The goodness-of-fit calculation is displayed in Table 7, with a GoF value of 0.54, which is significantly above 0.36; we can confirm the large goodness-of-fit of the model [12].

**Table 7:** Inner model assessment based on the goodness-of-fit of the model.

Latent variable	R Square	AVE	GOF
ENE		0.63	$\text{GoF}=\;\sqrt{\bar{R^{2}}\times \bar{\text{AVE}}}=0.54$
SON		0.59	
ATE	0.44	0.77	
ENC	0.40	0.61	
SEI	0.46	0.71	

**Fig. 4:**
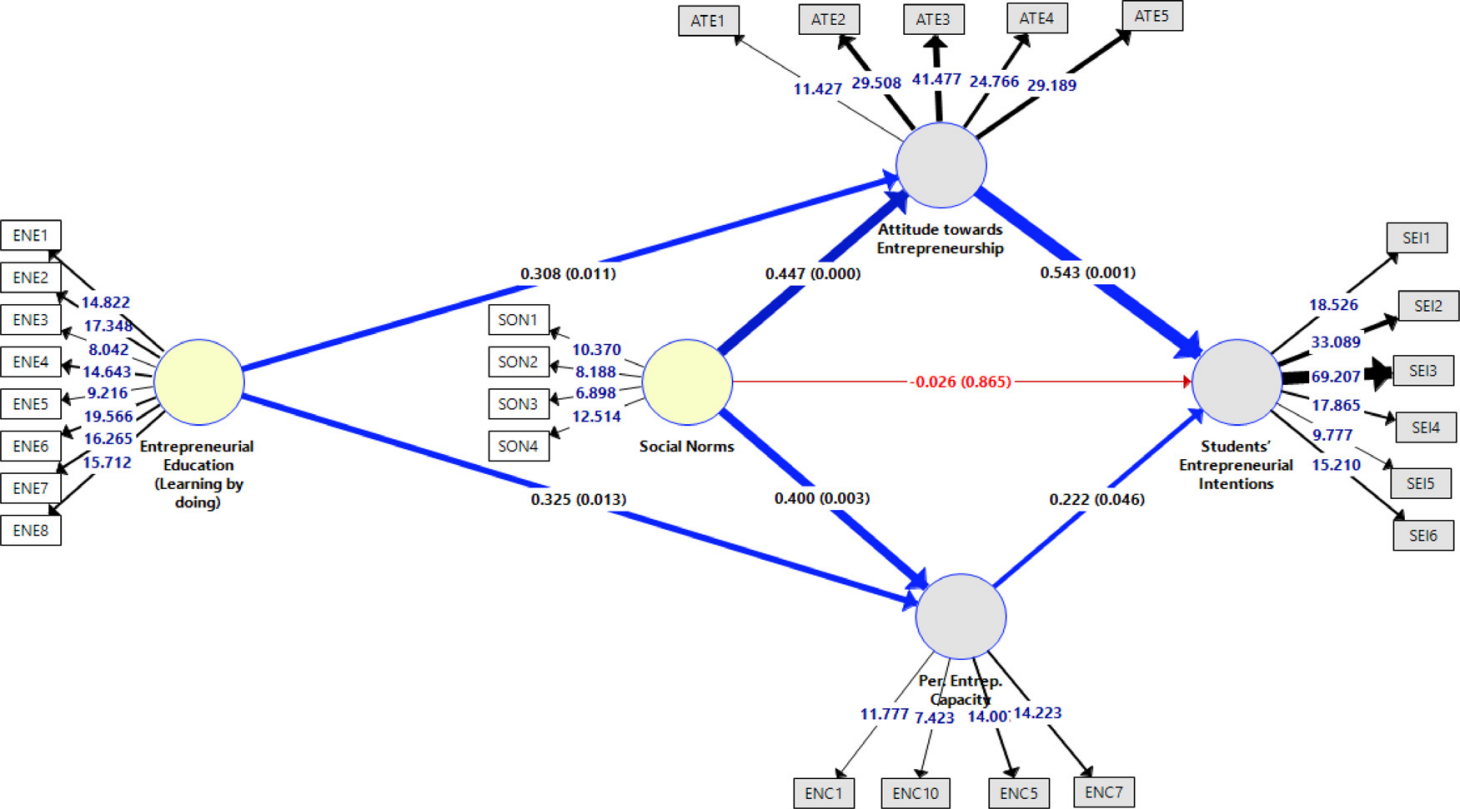
Inner model assessment.

As indicated in Table 8, the findings show that entrepreneurial education significantly influence on attitude towards entrepreneurship (ENE→ ATE: β-value= 0.308; p-value= 0.011), and perceived entrepreneurial capacity (ENE→ ENC: β-value= 0.325; p-value= 0.013). Furthermore, social norms positively impact on attitude towards entrepreneurship (SON→ ATE: β-value= 0.447; p-value= 0.000), and perceived entrepreneurial capacity (SON→ ENC: β-value= 0.400; p-value= 0.003). In addition, attitude towards entrepreneurship (ATE→ SEI: β-value= 0.543; p-value= 0.001), and perceived entrepreneurial capacity (ENC→ SEI: β= 0.222; p-value= 0.046) significantly and positively influence on students’ entrepreneurial intentions. However, the association between social norms and students’ entrepreneurial intentions (SON→ SEI: p-value= 0.865) were found to be not significant (Fig. 4).

**Table 8:** Inner model assessment - Hypotheses testing.

Hypotheses				Original Sample	Sample Mean	Standard Deviation	T Statistics	P Values	Outputs
H_1_	ENE	→	ATE	0.308	0.315	0.121	2.536	0.011	Accepted
H_2_	ENE	→	ENC	0.325	0.319	0.131	2.481	0.013	Accepted
H_3_	SON	→	ATE	0.447	0.443	0.120	3.716	0.000	Accepted
H_4_	SON	→	ENC	0.400	0.405	0.133	3.011	0.003	Accepted
H_5_	SON	→	SEI	-0.026	-0.039	0.152	0.170	0.865	Rejected
H_6_	ATE	→	SEI	0.543	0.568	0.156	3.481	0.001	Accepted
H_7_	ENC	→	SEI	0.222	0.212	0.112	1.992	0.046	Accepted

## Declaration of Competing Interest

The authors declare that they have no known competing financial interests or personal relationships that could have appeared to influence the work reported in this paper.

## References

[1] Cudeck R., Tinsley H.E.A., Brown S.D. (2000). Handb. Appl. Multivar. Stat. Math. Model..

[2] Hair J.F., Risher J.J., Sarstedt M., Ringle C.M. (2019). When to use and how to report the results of PLS-SEM. Eur. Bus. Rev..

[3] Bachiri M. (2016). Les déterminants de l'intention entrepreneuriale des étudiants, quels enseignements pour l'université marocaine?. Manag. Avenir..

[4] Boissin J.-P., Favre-Bonté V., Fine-Falcy S. (2017). Diverse impacts of the determinants of entrepreneurial intention: three submodels, three student profiles. Rev. L'Entrepreneuriat..

[5] Boissin J., Chollet B., Emin S. (2009). Les déterminants de l'intention de créer une entreprise chez les étudiants : un test empirique. M@n@gement.

[6] Liñán F., Rodríguez-Cohard J.C., Rueda-Cantuche J.M. (2011). Factors affecting entrepreneurial intention levels: a role for education. Int. Entrep. Manag. J..

[7] Adekiya A.A., Ibrahim F. (2016). Entrepreneurship intention among students. The antecedent role of culture and entrepreneurship training and development. Int. J. Manag. Educ..

[8] Boubker O., Arroud M., Ouajdouni A. (2021). Entrepreneurship education versus management students’ entrepreneurial intentions. A PLS-SEM approach. Int. J. Manag. Educ..

[9] Fornell C., Larcker D.F. (1981). Evaluating Structural Equation Models with Unobservable Variables and Measurement Error. J. Mark. Res..

[10] Henseler J., Ringle C.M., Sarstedt M. (2015). A new criterion for assessing discriminant validity in variance-based structural equation modeling. J. Acad. Mark. Sci..

[11] Hair J.F., Howard M.C., Nitzl C. (2020). Assessing measurement model quality in PLS-SEM using confirmatory composite analysis. J. Bus. Res..

[12] Boubker O., Douayri K. (2020). Dataset on the relationship between consumer satisfaction, brand attitude, brand preference and purchase intentions of dairy product: the case of the Laayoune-Sakia El Hamra region in Morocco. Data Brief.

